# Assessing the association between optimal energy intake and all‐cause mortality in older patients with diabetes mellitus using the Japanese Elderly Diabetes Intervention Trial

**DOI:** 10.1111/ggi.13820

**Published:** 2019-12-10

**Authors:** Takuya Omura, Yoshiaki Tamura, Takuya Yamaoka, Yukio Yoshimura, Takashi Sakurai, Hiroyuki Umegaki, Chiemi Kamada, Satoshi Iimuro, Yasuo Ohashi, Hideki Ito, Atsushi Araki, The Japanese Elderly Diabetes Intervention Trial Research Group

**Affiliations:** ^1^ Department of Diabetes, Metabolism and Endocrinology Tokyo Metropolitan Geriatric Hospital Tokyo Japan; ^2^ Training Department of Administrative Dietitians, Faculty of Human Life Science Shikoku University Tokushima Japan; ^3^ Center for Comprehensive Care and Research on Demented Disorders National Center for Geriatrics and Gerontology Aichi Japan; ^4^ Department of Community Healthcare and Geriatric Medicine Nagoya University Nagoya Japan; ^5^ Innovation and Research Support Center International University of Health and Welfare Tokyo Japan; ^6^ Department of Integrated Science and Engineering for Sustainable Society Chuo University Tokyo Japan

**Keywords:** bodyweight, diet therapy, elderly diabetes mellitus, energy intake, mortality

## Abstract

**Aim:**

Selecting optimal energy intake during diet therapy for older patients with diabetes mellitus is difficult because of the large differences in physical function and comorbid diseases. In Japan, although requirements for total energy intake are calculated by multiplying a person's *standard* bodyweight (BW) by the amount of physical activity, evidence supporting the application of this method among older people is limited. Therefore, we aimed to assess optimal energy intake by evaluating the relationship between energy intake and mortality in older patients.

**Methods:**

We evaluated data from a 6‐year prospective follow up of 756 older patients with diabetes mellitus, and the association between baseline nutrient intake and mortality. Total energy intake and nutrients were evaluated, and energy intake per *actual* BW was categorized into quartiles (Q). Cox regression analysis was used for statistical analyses. Energy intake per *standard* BW or age‐related *target* BW was statistically analyzed using the same protocol.

**Results:**

Analysis of energy intake per *actual* BW showed that hazard ratios for mortality was significantly higher in Q1 and Q4. Similar associations were found for energy intake per *standard* or *target* BW. Subgroup analysis showed that mortality rate was the lowest in Q2 in the young‐old population and in Q3 in the old‐old population.

**Conclusions:**

A U‐shaped relationship was observed between energy intake per BW and mortality in older patients with diabetes mellitus, which suggests that the optimal energy intake per *actual* or *target* BW should encompass a wide range to prevent malnutrition and excessive nutrition in these patients. **Geriatr Gerontol Int 2020; 20: 59–65**.

## Introduction

The aim of diet therapy in patients with diabetes mellitus includes sufficient intake of the nutrients required for daily living and improvement of abnormal glucose metabolism. Appropriate energy intake and having a balanced diet are essential for these patients. The Japan Diabetes Society (JDS) has defined the method by which standard bodyweight (BW) is calculated as (height [m])^2^ × 22 kg/m^2^, and ideal energy intake as standard BW × amount of physical activity (kcal/kg of standard BW based on light work, 25–30; moderate work, 30–35; heavy work, ≥35).^1^

The formula for calculating standard BW is determined by the relationship between the body mass index (BMI) and morbidity rate, which was obtained from the results of an epidemiological study of people aged 30–59 years;^2^ however, there is limited evidence from epidemiological studies on people aged >60 years. According to pooled analysis of the data from the Japan Diabetes Complications Study and the Japanese Elderly Diabetes Intervention Trial (J‐EDIT), which involved patients aged ≥75 years, it was observed that mortality risk was significantly higher in diabetes patients with BMI <18.5 kg/m^2^, and that this risk was lower in those with BMI ≈25 kg/m^2^.^3^ Therefore, the optimal BMI of older people might be different from the 22 kg/m^2^ proposed by the JDS. Standard BW is possibly lower than ideal in such people because of height loss with increasing age. Considering the relatively high optimal BMI of older people, age‐related target BW determined by (height [m])^2^ × 22–25 kg/m^2^ dependent on age rather than standard BW might be efficient for estimating energy requirement. Another method for estimating the energy requirement is to use the Harris‐Benedict equation; the method uses the resting energy expenditures (calculated from actual BW, height, and age) multiplied by activity factor and stress factor.^4^

Physical characteristics in older patients with diabetes mellitus vary according to age, obesity, sarcopenia, frailty, activities of daily living (ADL), amount of physical activity and comorbidities. Because diabetes mellitus is associated with prevalent and incident frailty, sufficient nutritional intake, including protein, to increase energy and prevent frailty is proposed even in older patients with diabetes mellitus.^5^, ^6^, ^7^ Malnutrition is common among older people with a lower BMI, and the proportions of the Japanese population with BMI <20 kg/m^2^ in men and women were 12.5% and 19.6%, respectively.^8^ Taken together, the optimal energy intake should be determined based on patient characteristics; however, this optimum value has never been identified because of insufficient scientific evidence. Based on this, determining the optimal energy intake is essential, and evaluating which calculation for energy intake (i.e. using standard, actual or target BW) is reasonable among older patients with diabetes mellitus. The present 6‐year prospective study aimed to assess the optimal energy intake by evaluating the relationship between energy intake and mortality in older Japanese adults with diabetes mellitus.

## Methods

### *Study design and participants*

In the current study, we evaluated data from 756 patients with diabetes mellitus (346 men) who were registered with J‐EDIT and evaluated for a baseline nutritional status. The J‐EDIT study was a prospective, randomized, controlled, multicenter intervention trial that evaluated 1173 patients from 39 institutions. These patients had diabetes, were aged ≥65 years and had glycated hemoglobin (HbA1c) ≥7.9% or ≥7.4% with at least one cardiovascular risk factor. When time‐to‐event analysis was carried out, patients who dropped out during the follow‐up period (*n* = 104) and those with insufficient file information (e.g. missing data or lacking nutrition information; *n* = 313) were excluded from the analysis. Details regarding the J‐EDIT protocol have been published elsewhere.^9^ Diabetes mellitus was diagnosed according to the Report of the Committee of the JDS on the Classification and Diagnostic Criteria of Diabetes Mellitus.^10^

Information on the history of ischemic heart disease (IHD) and stroke was obtained from medical records. IHD was considered to be present when the patients showed at least one of the following: (i) a history of myocardial infarction, typical electrocardiography alteration and enzymatic changes (creatine kinase [CK], CK‐MB [myocardial band]); and (ii) a history of angina pectoris and positive post‐load electrocardiography or cardiac scintigram findings confirmed by coronary angiography. Stroke was defined as clinical sign of a focal neurological deficit with rapid onset that persisted for 24 h confirmed by the findings of either brain computed tomography or magnetic resonance imaging. No cases of asymptomatic lesions detected by brain imaging were included.

The frequency of mild or severe hypoglycemia was assessed based on questionnaires that included questions, such as the number of times the patient had hypoglycemic episodes in a year, month or week and the number of times the patient experienced a coma, or had an emergency visit or were admitted to a hospital because of hypoglycemia in a year, month or week. Mild hypoglycemia episodes were defined as those that included both appearance and recovery of hypoglycemic symptoms after ingesting glucose or sugar. The episodes of severe hypoglycemia were defined as those during which the patient experienced a coma or convulsions, or required the assistance of another person for recovery.

Written informed consent was obtained from all of the participants. For some patients recognized as cognitively impaired, we obtained proxy consent from a family member or another supportive adult on their behalf. This study was approved by the ethics committee of Tokyo Metropolitan Geriatric Hospital.

### *Clinical and laboratory measurements*

The clinical and laboratory assessments of the patients were carried out at least annually after the baseline evaluation. Mean values for at least two measurements each year were obtained for HbA1c, fasting plasma glucose and fasting serum lipids. HbA1c assays were carried out according to procedures outlined by the Laboratory Test Committee of JDS, which is converted by HbA1c (National Glycohemoglobin Standardization Program) (%) = 1.02 × HbA1c (JDS) (%) + 0.25%. Serum low‐density lipoprotein (LDL) cholesterol was calculated using the Friedewald equation. Estimated glomerular filtration rate (eGFR) was calculated according to the Japanese coefficient‐modified Modification of Diet in Renal Disease Study equation as follows^11^: eGFR (mL/min/1.73 m^2^) = 194 × (serum creatinine) ^−1.094^ × (age) ^− 0.287^ (× 0.739, if female). All other measurements, including those for BW and blood pressure, were taken at least once annually.

### *Assessment of nutrition and physical activities*

Nutritional intake for 1 week at baseline was assessed using a Yoshimura's food frequency questionnaire based on food groups.^12^ This 46‐item questionnaire describes a standard portion size for each food item, and patients are asked to select the portion size that they typically consume (50%, 100% or 200% of a standard portion) and the intake frequency per week for each food. The validated Yoshimura's food frequency questionnaire method allowed us to estimate food and total energy intake; carbohydrate‐to‐energy ratio; protein‐to‐energy ratio; fat‐to‐energy ratio; and cholesterol, salt, iron, calcium, vitamin, and dietary fiber intake on the basis of portion size (relative to the standard amount) and frequency (times eaten in 1 week) of 29 food groups using standardized software for population‐based surveys and nutritional counseling in Japan (Excel EIYO‐Kun, v. 4.5; Shikoku University Nutritional Database, Kenpakusha, Japan).

Three indices of energy intake were calculated by dividing the total energy intake by actual, standard and target weights. Target BW was set to 23.5 kg/m^2^  × (height [m])^2^ for patients aged <75 years, and 25.0 kg/m^2^ × (height [m])^2^ for those aged ≥75 years. The coefficients were determined with reference to the target BMI of 20.0–24.9 (~22.5) for people aged 50–69 years, and 21.5–24.9 (~23.2) for those aged ≥70 years in the Dietary Reference Intakes for Japanese released by the Ministry of Health, Labor and Welfare (2017).^8^

Physical activity was assessed using Baecke's questionnaire.^13^ ADL was evaluated using the 13‐item Tokyo Metropolitan Institute of Gerontology Index of Competence, which includes instrumental ADL, intellectual activity and social roles.^14^

### *End‐points*

The end‐point in the present study was all‐cause mortality. Fatal and non‐fatal events identified during the follow‐up period were certified by at least two members of the experts’ committee, who were masked for risk factor status, and another member's diagnosis. When an event was noted on the annual data sheet, the administrator requested full information from the database center, which was reviewed by two clinical assessors of the event assignment committee. These two separate assessments for each event were entered on special data sheets. If there was disagreement on the assessment, the final decision was made by discussion in a meeting of event assignment committee members.

### *Statistical analysis*

Data from J‐EDIT are described as the mean ± SD or median within the interquartile range. Hazard ratios (HR) for mortality among quartiles categorized by energy intake per actual BW (kg) per day (Q1, ≤24.85 kcal/kg BW/day; Q2, 24.86–29.73 kcal/kg BW/day; Q3, 29.74–34.78 kcal/kg BW/day; and Q4, ≥34.79 kcal/kg BW/day) were estimated using Cox regression analysis. Analyses were carried out using the following models: the crude model (model 1); adjusted for age, sex and BMI (model 2); adjusted for age, sex, BMI, HbA1c, systolic blood pressure, LDL cholesterol, eGFR, physical activity, history of IHD, history of stroke and history of hypoglycemia (model 3); and adjusted for age, sex, BMI, HbA1c, systolic blood pressure, LDL cholesterol, eGFR, physical activity, history of IHD, history of stroke, history of hypoglycemia and protein intake per actual BW (model 4). An adjustment for protein intake was added to model 4, because increasing protein intake was associated with increasing energy intake. Patients with missing data in the analyses were excluded (complete case analysis).

All statistical analyses were carried out using spss version 25 (IBM Corporation, Armonk, NY, USA). In all comparisons, the significance level was set at *P* < 0.05.

## Results

The baseline characteristics of 756 patients are presented in Table 1. Energy intake per actual BW/day was categorized into quartile groups. Mean total energy intake across quartiles ranged from 1397 to 2086 kcal/day. In each group, no significant differences were observed in age, systolic blood pressure and LDL cholesterol. As energy intake per actual BW increased, BMI tended to decrease, whereas physical activity, total energy intake and protein intake tended to increase.

**Table 1:** Baseline clinical characteristics of 756 patients with diabetes mellitus according to quartiles of energy intake per *actual* bodyweight

	Q1	Q2	Q3	Q4	*P*‐value*
	≤24.85	24.86–29.73	29.74–34.78	≥34.79	
	kcal/kg BW	kcal/kg BW	kcal/kg BW	kcal/kg BW	
	(*n* = 189)	(*n* = 189)	(*n* = 189)	(*n* = 189)	
Age (years)	71.1 ± 4.4	71.9 ± 4.8	72.1 ± 4.9	72.1 ± 4.8	0.10
Women (%)	46.0	51.9	56.6	62.4	0.01
Height (cm)	158 ± 8.7	157 ± 9.0	155 ± 8.4	154 ± 8.2	<0.01
Bodyweight (kg)	64.8 ± 9.4	59.8 ± 8.9	55.9 ± 7.8	51.4 ± 8.5	<0.01
Body mass index (kg/m^2^)	26.1 ± 3.5	24.3 ± 2.8	23.1 ± 2.7	21.7 ± 2.8	<0.01
Systolic blood pressure (mmHg)	136 ± 15	138 ± 16	135 ± 15	135 ± 16	0.15
HbA1c (%)	8.1 ± 1.1	8.0 ± 0.8	7.9 ± 0.7	8.1 ± 0.9	0.04
History of diabetic nephropathy (%)	47.6	48.4	43.9	40.7	0.41
eGFR (mL/min/1.73 m^2^)	63.8 ± 19.7	65.6 ± 19.5	66.3 ± 15.5	69.7 ± 21.2	0.03
LDL cholesterol (mg/dL)	121 ± 34	123 ± 31	120 ± 29	116 ± 30	0.13
Physical activity (Baecke)	7.2 ± 3.2	7.7 ± 2.7	7.8 ± 3.1	8.4 ± 2.9	<0.01
ADL (TMIG Index)	11.4 ± 2.4	11.8 ± 1.8	11.8 ± 2.0	11.9 ± 1.8	0.06
MMSE	27.9 ± 2.8	27.8 ± 2.5	28.1 ± 2.4	28.3 ± 2.1	0.35
Total energy intake (kcal/day)	1397 ± 223	1629 ± 250	1792 ± 246	2086 ± 361	<0.01
Indicated energy amount (kcal/day)	1487 ± 197	1490 ± 203	1460 ± 196	1458 ± 182	0.26
Protein intake (g/day/kg BW)	1.0 ± 0.2	1.1 ± 0.2	1.2 ± 0.3	1.4 ± 0.3	<0.01
Protein energy ratio (%)	14.8 ± 2.2	15.3 ± 2.0	15.7 ± 2.0	16.0 ± 2.2	<0.01
Carbohydrate intake (g/day/kg BW)	3.8 ± 0.6	4.1 ± 0.7	4.5 ± 0.8	4.9 ± 1.0	<0.01
Carbohydrate energy ratio (%)	61.8 ± 5.8	59.7 ± 5.3	58.5 ± 5.2	56.3 ± 5.8	<0.01
Fat intake (g/day/kg BW)	0.8 ± 0.2	0.8 ± 0.2	0.9 ± 0.2	1.0 ± 0.3	<0.01
Fat energy ratio (%)	23.5 ± 4.5	25.0 ± 4.3	25.8 ± 4.3	27.7 ± 4.5	<0.01
All‐cause death	25	11	8	15	<0.01
Cardiovascular death	7	6	3	2	0.28
Cancer death	9	1	5	5	0.09

Data are the mean ± standard deviation or *n* (%).

* *P*‐value for one‐way analysis of variance.

ADL, activities of daily living; BW, bodyweight; eGFR, estimated glomerular filtration rate; HbA1c, glycated hemoglobin; LDL, low‐density lipoprotein; MMSE, Mini‐Mental State Examination; Q, quartile; TMIG Index, Tokyo Metropolitan Institute of Gerontology Index of Competence.

During the 6‐year follow‐up period, there were 59 cases of all‐cause death (18 from cardiovascular disease, including sudden death; 20 from cancer; and 21 from the other diseases). Table 2 shows HR of all‐cause mortality among the quartile groups categorized by energy intake per actual and standard BW for all four models. The analysis of energy intake per actual BW showed that HR for mortality either was or tended to be significantly higher in Q1 and Q4, respectively, than in the Q3 group as a reference (models 1 and 2). The mortality risk in Q2 and Q4 was attenuated as covariables were further adjusted (model 3). After additional adjustment for protein intake, the significant association persisted between low energy intake and mortality rate (model 4). When we entered the intake of fat or dietary fibers instead of protein into the model, similar associations between energy intake and mortality remained (Table S1). Similarly, in the analysis of the association between energy intake per standard BW and death, the mortality rate was lowest in Q3 across all models.

**Table 2:** Cox regression analysis of quartiles of energy intake per *actual* or *standard* bodyweight and all‐cause mortality

*Actual* BW									
	Events	Q1		Q2		Q3	Q4		*P*‐value
		≤24.85		24.86–29.73		29.74–34.78	≥34.79		
		kcal/kg BW		kcal/kg BW		kcal/kg BW	kcal/kg BW		
		HR	*P*	HR	*P*	Reference	HR	*P*	
		(95% CI)		(95% CI)			(95% CI)		
Model 1 (crude)	59	3.21	0.004	1.40	0.473	1	1.91	0.141	0.051
		(1.45–7.12)		(0.56–3.47)			(0.81–4.49)		
Model 2†	59	4.16	0.001	1.56	0.342	1	1.77	0.198	0.011
		(1.80–9.58)		(0.62–3.92)			(0.74–4.21)		
Model 3‡	50	3.87	0.002	0.94	0.905	1	1.54	0.355	0.016
		(1.63–9.16)		(0.32–2.76)			(0.62–3.82)		
Model 4§	50	3.83	0.002	0.92	0.883	1	1.60	0.313	0.020
		(1.62–9.09)		(0.31–2.72)			(0.64–4.00)		
*Standard* BW									
	Events	Q1		Q2		Q3	Q4		*P*‐value
		≤27.77		27.78–31.44		31.45–36.42	≥36.43		
		kcal/kg BW		kcal/kg BW		kcal/kg BW	kcal/kg BW		
		HR	*P*	HR	*P*	Reference	HR	*P*	
		(95% CI)		(95% CI)			(95% CI)		
Model 1 (crude)	59	3.23	0.004	1.21	0.689	1	1.83	0.166	0.042
		(1.46–7.14)		(0.48–3.06)			(0.78–4.33)		
Model 2†	59	3.57	0.002	1.51	0.387	1	2.20	0.074	0.049
		(1.60–7.97)		(0.59–3.86)			(0.93–5.21)		
Model 3‡	50	3.94	0.004	1.68	0.334	1	2.22	0.116	0.046
		(1.57–9.94)		(0.59–4.79)			(0.82–6.00)		
Model 4§	50	3.88	0.004	1.67	0.337	1	2.23	0.114	0.054
		(1.53–9.79)		(0.59–4.78)			(0.83–6.03)		

† Adjusted for age, sex and body mass index.

‡ Adjusted for age, sex, body mass index, glycated hemoglobin, systolic blood pressure, low‐density lipoprotein cholesterol, estimated glomerular filtration rate, physical activity, history of ischemic heart disease, history of stroke and history of hypoglycemia.

§ Adjusted for age, sex, body mass index, glycated hemoglobin, systolic blood pressure, low‐density lipoprotein cholesterol, estimated glomerular filtration rate, physical activity, history of ischemic heart disease, history of stroke, history of hypoglycemia and protein intake per actual bodyweight. CI, confidence interval; HR, hazard ratio; Q, quartile.

Table 3 shows HR of mortality among quartiles categorized by energy intake per target BW. The results showed that the mortality rate was lowest in Q3 in target BW calculated from 23.5 kg/m^2^× (height [m])^2^ for patients aged <75 years, and 25.0 kg/m^2^× (height [m])^2^ for those aged ≥75 years.

**Table 3:** Cox regression analysis of quartiles of energy intake per *target* bodyweight and all‐cause mortality

*Target* BW									
	Events	Q1		Q2		Q3	Q4		*P*‐value
		≤25.38		25.39–29.03		29.04–34.72	≥34.73		
		kcal/kg BW		kcal/kg BW		kcal/kg BW	kcal/kg BW		
		HR	*P*	HR	*P*	Reference	HR	*P*	
		(95% CI)		(95% CI)			(95% CI)		
Model 1 (crude)	59	2.70	0.006	1.12	0.783	1	1.54	0.302	0.040
		(1.33–5.48)		(0.49–2.59)			(0.68–3.49)		
Model 2†	59	2.56	0.010	1.22	0.637	1	1.82	0.153	0.113
		(1.25–5.23)		(0.53–2.83)			(0.80–4.15)		
Model 3‡	50	2.70	0.014	1.16	0.749	1	1.58	0.341	0.064
		(1.22–5.98)		(0.46–2.96)			(0.62–4.04)		
Model 4§	50	2.64	0.017	1.15	0.765	1	1.58	0.337	0.076
		(1.19–5.88)		(0.45–2.93)			(0.62–4.05)		

23.5 kg/m^2^ × (height [m])^2^ for patients aged <75 years, and 25.0 kg/m^2^ × (height [m])^2^ for those aged ≥75 years.

† Adjusted for age, sex and body mass index.

‡ Adjusted for age, sex, body mass index, glycated hemoglobin, systolic blood pressure, low‐density lipoprotein cholesterol, estimated glomerular filtration rate, physical activity, history of ischemic heart disease, history of stroke and history of hypoglycemia.

§ Adjusted for age, sex, body mass index, glycated hemoglobin, systolic blood pressure, low‐density lipoprotein cholesterol, estimated glomerular filtration rate, physical activity, history of ischemic heart disease, history of stroke, history of hypoglycemia and protein intake per actual bodyweight. CI, confidence interval; HR, hazard ratio; Q, quartile.

Subgroup analyses were carried out for energy intake per actual BW according to sex and age (patients aged <75 years and those aged ≥75 years; Table 4). The results showed that the mortality rate was lowest in Q2 in men and Q3 in women. In addition, the mortality rate was lowest in Q2 in patients aged <75 years, and in Q3 in those aged ≥75 years.

**Table 4:** Subgroup analyses of the association among quartiles of energy intake per *actual* bodyweight and all‐cause mortality in patients with diabetes mellitus

	Events	Q1		Q2		Q3	Q4		*P*‐value
		≤24.85		24.86–29.73		29.74–34.78	≥34.79		
		kcal/kg BW		kcal/kg BW		kcal/kg BW	kcal/kg BW		
		HR	*P*	HR	*P*	Reference	HR	*P*	
		(95% CI)		(95% CI)			(95% CI)		
**Men**									
Model 1 (crude)	36	3.37	0.016	0.95	0.930	1	1.71	0.359	0.042
		(1.26–9.04)		(0.27–3.27)			(0.54–5.39)		
Model 2†	36	3.99	0.008	0.97	0.960	1	1.51	0.484	0.016
		(1.44–11.1)		(0.28–3.38)			(0.48–4.78)		
Model 3‡	30	3.78	0.014	0.42	0.302	1	1.22	0.761	0.016
		(1.31–10.9)		(0.08–2.20)			(0.35–4.29)		
Model 4§	30	3.81	0.014	0.42	0.305	1	1.20	0.778	0.016
		(1.32–11.0)		(0.08–2.21)			(0.34–4.25)		
**Women**									
Model 1 (crude)	23	2.34	0.230	2.16	0.278	1	2.40	0.195	0.857
		(0.59–9.35)		(0.54–8.62)			(0.64–9.06)		
Model 2†	23	3.76	0.080	2.88	0.140	1	2.23	0.247	0.323
		(0.85–16.6)		(0.71–11.8)			(0.57–8.64)		
Model 3‡	20	4.20	0.070	2.32	0.289	1	1.80	0.414	0.228
		(0.89–19.8)		(0.49–11.0)			(0.44–7.31)		
Model 4§	20	4.21	0.074	2.14	0.338	1	1.96	0.351	0.303
		(0.87–20.4)		(0.45–10.1)			(0.48–8.05)		
									
**Age <75 years**									
Model 1 (crude)	34	2.95	0.035	0.95	0.930	1	1.66	0.375	0.117
		(1.08–8.06)		(0.27–3.27)			(0.54–5.07)		
Model 2†	34	3.13	0.032	0.94	0.928	1	1.75	0.332	0.149
		(1.10–8.89)		(0.27–3.28)			(0.56–5.46)		
Model 3‡	30	2.97	0.046	0.62	0.516	1	1.43	0.572	0.100
		(1.02–8.63)		(0.15–2.63)			(0.41–5.01)		
Model 4§	30	2.98	0.045	0.63	0.525	1	1.42	0.584	0.092
		(1.02–8.70)		(0.15–2.66)			(0.41–4.98)		
**Age ≥75 years**									
Model 1 (crude)	25	4.31	0.028	2.51	0.194	1	2.28	0.234	0.115
		(1.17–15.9)		(0.63–10.0)			(0.59–8.80)		
Model 2†	25	6.30	0.010	2.88	0.140	1	1.81	0.398	0.028
		(1.57–25.3)		(0.71–11.8)			(0.46–7.16)		
Model 3‡	20	6.19	0.022	1.56	0.609	1	1.38	0.669	0.050
		(1.30–29.6)		(0.28–8.56)			(0.31–6.12)		
Model 4§	20	7.88	0.012	1.88	0.472	1	2.26	0.315	0.074
		(1.59–39.1)		(0.34–10.5)			(0.46–11.1)		

† Adjusted for age, sex and body mass index.

‡ Adjusted for age, sex, body mass index, glycated hemoglobin, systolic blood pressure, low‐density lipoprotein cholesterol, estimated glomerular filtration rate, physical activity, history of ischemic heart disease, history of stroke and history of hypoglycemia.

§ Adjusted for age, sex, body mass index, glycated hemoglobin, systolic blood pressure, low‐density lipoprotein cholesterol, estimated glomerular filtration rate, physical activity, history of ischemic heart disease, history of stroke, history of hypoglycemia and protein intake per actual body weight. CI, confidence interval; HR, hazard ratio; Q, quartile.

## Discussion

In the present study, the change in energy intake/day was approximately 1.5‐fold between the group with the highest energy and that with the lowest (i.e. Q4 *vs* Q1), with the mean for each group within a range of ~1400–2000 kcal. In contrast, the indicated energy intake/day of each group was within a range of 1458–1490 kcal (Table 1, S3). This result suggests that older patients really take excessive energy intake, although they are likely to underreport dietary content when filling out the self‐report form, similar to that in other studies.

We investigated the relationship between energy intake per actual BW and all‐cause mortality rate (Table 2; Fig. S1). The results showed that the HR for mortality was highest in Q1 and Q4, and lowest in Q3 in models 1 and 2 or in Q2 in models 3 and 4. The U‐shaped relationship between energy intake per actual BW and mortality was observed in all adjusted models, including ADL or cognition (Table S2). This U‐shaped curve was also found even after subgroup analyses according to BMI value (data not shown). The result of the association between low energy intake and high mortality is consistent with that of previous reports in an older general population.^15^, ^16^ In contrast, the relationship between high‐energy intake and increased mortality was observed in another study that included young men.^17^ To the best of our knowledge, this is the first study showing the U‐shaped association between energy intake and mortality in older patients with diabetes. Considering the results in models 3 and 4, the optimal energy intake per actual BW would have a wide range of between 25 and 35 kcal/kg BW.

Patients in the Q1 group showed the highest BMI value, although their energy and protein intake, and amount of physical activity were the lowest. This suggests that patients with sarcopenic obesity might be included in this group, and that BMI did not reflect the actual intake of energy and nutrients in older patients. Alternatively, obese patients in the Q1 group might have underreported the dietary intake. When Cox regression analyses including BMI were carried out, the association between low energy intake and mortality persisted. The U‐shaped curve was also found in the subgroup analyses according to BMI value (data not shown). Collectively, energy intake per bodyweight might have greater effects on mortality than BMI.

The analysis of energy intake per standard BW showed that HR for mortality was lowest in Q3 at 31.45–36.42/kg BW, which was similar to the coefficients of moderate work (30–35 kcal/kg) described in the guidelines of physical activity. Physical activity tends to decrease with increasing age. Given that decrease, energy intake calculated from standard BW multiplied by the coefficient of light work (25–30 kcal/kg), which reflects the physical activity of most of older patients, could have been lower than it should have been.

**Figure 1:**
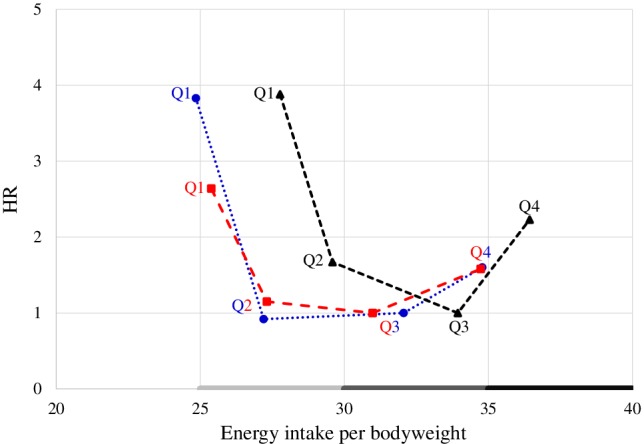
Graphical scheme of the relationship between the hazard ratio (HR) for mortality and energy intake per *actual*, *standard* or *target* bodyweight. The HR is plotted on the *y*‐axis and energy intake per *actual* (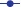), *standard* (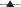) or *target* (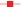) bodyweight is plotted on the *x*‐axis using model 4. Q, quartile.

We also used energy intake per target BW, which was calculated from age‐specific coefficients using 23.5 kg/m^2^× (height [m])^2^ for patients aged <75 years, and 25.0 kg/m^2^ × (height [m])^2^ for those aged ≥75 years, which was heavier than the standard BW (Table 3). The mortality rate was lowest in Q3, with an energy intake per target BW of 29.04–34.72 kcal/kg BW. The energy intake per target or actual BW with the lowest mortality risk shifted to the left when compared with that per standard BW (Fig. 1).

The association between energy intake and mortality might be affected by the intake of macronutrients and dietary fibers. The intake of high protein in young people and low protein intake in older people^18^ and high carbohydrate intake in the general population^19^ were associated with mortality. The low intake of dietary fiber was associated with mortality in patients with diabetes mellitus.^20^ In the present study, the similar association between low energy intake and mortality persisted after adjusting the intake of protein (model 4), carbohydrate, fat or dietary fiber (Table S1); therefore, it would be extremely important to ensure sufficient energy intake for survival independent of protein or dietary fiber intake in older patients with diabetes mellitus.

A study showed that lower BMI was associated with an increased risk of death in patients with diabetes mellitus, and such risk was even higher in those aged ≥75 years;^3^ therefore, we carried out subgroup analyses according to age and sex (Table 4). In each analysis, we described the relationship between energy intake per actual BW and HR for mortality, which showed a U‐shaped curve. The mortality rate was lowest in Q2 among patients aged 65–74 years, and in Q3 among those aged ≥75 years. The present study also suggested that energy intake greater than that conventionally recommended might be desirable among old‐old diabetes patients.

The present study had some limitations. First, because it was a 6‐year prospective longitudinal study, the cause–effect relationship remains unknown; however, low energy intake in older patients could result in sarcopenia, frailty, ADL disability and infection because of low immune function, all of which lead to increased mortality. Second, we did not carry out an analysis on the relationship between energy intake and specific cause of death because of the small number of cases. Third, because the present study participants were recruited from the Japanese population >10 years ago, the results should be verified using the current Japanese and other populations. Finally, although we carried out several multivariate analyses, the confounding effect of other factors, such as muscle mass, muscle strength and socioeconomic conditions, cannot be neglected.

In conclusion, a U‐shaped relationship was observed between energy intake per actual BW and mortality in older patients with diabetes. Similar results were obtained in the analyses using the standard or age‐related target BW; however, when using the target or actual BW, the energy intake with the lowest mortality risk shifted to the left when compared with that using standard BW. The optimal energy intake per actual or target BW would be within a wide range of 25–35 kcal/kg BW depending on age and physical status. The present study also suggested that energy intake greater than that conventionally recommended might be necessary for older patients with diabetes mellitus. To obtain an energy intake greater than that conventionally recommended, using age‐related target BW would be better than using standard BW. Additional studies are necessary to examine what levels and components of energy intake could lead to better physical function, quality of life and survival.

## Disclosure statement

The authors declare no conflict of interest.

## Supporting information


**Table S1.** Association between dietary content and all‐cause mortality in patients with diabetes mellitus
**Table S2.** Cox regression analysis of quartiles of energy intake per actual bodyweight and all‐cause mortality in model 4, and the model further adjusted for activities of daily living and Mini‐Mental State Examination
**Table S3.**. Baseline clinical characteristics of 756 patients with diabetes mellitus according to quartiles of body mass index
**Figure S1.** Survival curve for Cox proportional hazards model
