# Supplementary material for: Assessing the association between optimal energy intake and all‐cause mortality in older patients with diabetes mellitus using the Japanese Elderly Diabetes Intervention Trial
Source: Geriatr Gerontol Int. 2019 Dec 10;20(1):59–65. doi: 10.1111/ggi.13820 (PMC7003876; doi:10.1111/ggi.13820)

**Supplementary Table 1. Association between dietary content and all–cause mortality in patients with diabetes mellitus**

| ***Actual* body weight** | | | |  | |  |  | |  |
| --- | --- | --- | --- | --- | --- | --- | --- | --- | --- |
|  | Events | **Q1** | | **Q2** | | **Q3** | **Q4** | | P–value |
|  |  | ≤24.85 | | 24.86–29.73 | | 29.74–34.78 | ≥34.79 | |  |
|  |  | kcal/kg BW | | kcal/kg BW | | kcal/kg BW | kcal/kg BW | |  |
|  |  | HR | p | HR | p | Reference | HR | p |  |
|  |  | (95%CI) |  | (95%CI) |  |  | (95%CI) |  |  |
| Model 1* | 50 | 3.83 | 0.002 | 0.92 | 0.883 | 1 | 1.60 | 0.313 | 0.020 |
|  |  | (1.62–9.09) |  | (0.31–2.72) |  |  | (0.64–4.00) |  |  |
| Model 2** | 50 | 3.84 | 0.002 | 0.93 | 0.893 | 1 | 1.56 | 0.343 | 0.017 |
|  |  | (1.62–9.11) |  | (0.31–2.74) |  |  | (0.62–3.89) |  |  |
| Model 3*** | 50 | 3.67 | 0.003 | 0.91 | 0.871 | 1 | 1.40 | 0.478 | 0.012 |
|  |  | (1.55–8.68) |  | (0.31–2.70) |  |  | (0.56–3.50) |  |  |
| Model 4 **** | 50 | 3.76 | 0.003 | 0.90 | 0.850 | 1 | 1.59 | 0.322 | 0.023 |
|  |  | (1.58–8.94) |  | (0.31–2.66) |  |  | (0.64–3.95) |  |  |
| ***Standard* body weight** | | | |  | |  |  | |  |
|  | Events | **Q1** | | **Q2** | | **Q3** | **Q4** | | P–value |
|  |  | ≤27.77 | | 27.78–31.44 | | 31.45–36.42 | ≥36.43 | |  |
|  |  | kcal/kg BW | | kcal/kg BW | | kcal/kg BW | kcal/kg BW | |  |
|  |  | HR | p | HR | p | Reference | HR | p |  |
|  |  | (95%CI) |  | (95%CI) |  |  | (95%CI) |  |  |
| Model 1* | 50 | 3.88 | 0.04 | 1.67 | 0.337 | 1 | 2.23 | 0.114 | 0.054 |
|  |  | (1.53–9.79) |  | (0.59–4.78) |  |  | (0.83–6.03) |  |  |
| Model 2** | 50 | 3.93 | 0.004 | 1.67 | 0.336 | 1 | 2.23 | 0.115 | 0.049 |
|  |  | (1.56–9.93) |  | (0.59–4.78) |  |  | (0.82–6.04) |  |  |
| Model 3*** | 50 | 4.05 | 0.003 | 1.70 | 0.321 | 1 | 2.11 | 0.143 | 0.031 |
|  |  | (1.60–10.2) |  | (0.60–4.84) |  |  | (0.78–5.71) |  |  |
| Model 4 **** | 50 | 3.82 | 0.005 | 1.63 | 0.361 | 1 | 2.25 | 0.110 | 0.064 |
|  |  | (1.51–9.67) |  | (0.57–4.66) |  |  | (0.83–6.08) |  |  |

* Adjusted for age, sex, BMI, HbA1c, SBP, LDL–cholesterol, eGFR, Physical activity, history of IHD, history of stroke, history of hypoglycemia, and protein intake per *actual* body weight

** Adjusted for Adjusted for age, sex, BMI, HbA1c, SBP, LDL–cholesterol, eGFR, Physical activity, history of IHD, history of stroke, history of hypoglycemia, and carbohydrate intake per *actual* body weight

*** Adjusted for Adjusted for age, sex, BMI, HbA1c, SBP, LDL–cholesterol, eGFR, Physical activity, history of IHD, history of stroke, history of hypoglycemia, and fat intake per *actual* body weight

**** Adjusted for age, sex, BMI, HbA1c, SBP, LDL–cholesterol, eGFR, Physical activity, history of IHD, history of stroke, history of hypoglycemia, and dietary fiber per *actual* body weight

**Supplementary Table 2.** **Cox regression analysis of quartiles of energy intake per *actual* body weight and all–cause mortality in the model 4 and model further adjusted for activity of daily life (ADL) and Mini–Mental State Examination (MMSE)**

| ***Actual* body weight** | | | |  | |  |  | |  |
| --- | --- | --- | --- | --- | --- | --- | --- | --- | --- |
|  | Events | **Q1** | | **Q2** | | **Q3** | **Q4** | | P–value |
|  |  | ≤24.85 | | 24.86–29.73 | | 29.74–34.78 | ≥34.79 | |  |
|  |  | kcal/kg BW | | kcal/kg BW | | kcal/kg BW | kcal/kg BW | |  |
|  |  | HR | p | HR | p | Reference | HR | p |  |
|  |  | (95%CI) |  | (95%CI) |  |  | (95%CI) |  |  |
| Model 1* | 50 | 3.83 | 0.002 | 0.92 | 0.883 | 1 | 1.60 | 0.313 | 0.020 |
|  |  | (1.62–9.09) |  | (0.31–2.72) |  |  | (0.64–4.00) |  |  |
| Model 2** | 50 | 3.58 | 0.004 | 0.92 | 0.881 | 1 | 1.64 | 0.289 | 0.035 |
|  |  | (1.51–8.46) |  | (0.31–2.72) |  |  | (0.66–4.11) |  |  |
| Model 3*** | 43 | 3.01 | 0.016 | 0.74 | 0.609 | 1 | 1.44 | 0.453 | 0.077 |
|  |  | (1.23–7.36) |  | (0.24–2.33) |  |  | (0.56–3.73) |  |  |
| Model 4 **** | 43 | 2.99 | 0.016 | 0.78 | 0.662 | 1 | 1.48 | 0.418 | 0.088 |
|  |  | (1.23–7.30) |  | (0.25–2.44) |  |  | (0.57–3.85) |  |  |

* Adjusted for age, sex, HbA1c, SBP, LDL–cholesterol, eGFR, Physical activity, history of IHD, history of stroke, history of hypoglycemia, and protein intake per *actual* body weight

** Adjusted for Adjusted for age, sex, HbA1c, SBP, LDL–cholesterol, eGFR, Physical activity, history of IHD, history of stroke, history of hypoglycemia, protein intake per *actual* body weight, and ADL

*** Adjusted for Adjusted for age, sex, HbA1c, SBP, LDL–cholesterol, eGFR, Physical activity, history of IHD, history of stroke, history of hypoglycemia, protein intake per *actual* body weight, and MMSE

**** Adjusted for age, sex, HbA1c, SBP, LDL–cholesterol, eGFR, Physical activity, history of IHD, history of stroke, history of hypoglycemia, protein intake per *actual* body weight, ADL, and MMSE

**Supplementary Table 3. Baseline clinical characteristics of 756 patients with diabetes mellitus according to quartiles of body mass index**

|  | **Q1** | **Q2** | **Q3** | **Q4** | P–value* |
| --- | --- | --- | --- | --- | --- |
|  | ≤21.5 | 21.6–23.4 | 23.5–25.6 | ≥25.7 |  |
|  | kg/m^2^ | kg/m^2^ | kg/m^2^ | kg/m^2^ |  |
|  | (n= 183) | (n= 191) | (n= 193) | (n= 189) |  |
| Age (years) | 71.9 ± 4.7 | 72.0 ± 4.9 | 71.8 ± 4.7 | 71.6 ± 4.7 | 0.84 |
| Women (%) | 54.1 | 48.2 | 52.8 | 61.9 | 0.06 |
| Height (cm) | 156 ± 8.6 | 157 ± 9.0 | 157 ± 8.3 | 154 ± 8.5 | <0.01 |
| Body weight (kg) | 48.4 ± 6.6 | 55.4 ± 6.6 | 60.1 ± 6.6 | 67.6 ± 8.8 | <0.01 |
| HbA1c (%) | 8.1 ± 1.0 | 8.0 ± 0.8 | 8.0 ± 0.9 | 8.1 ± 1.0 | 0.88 |
| eGFR (ml/min/1.73m^2^) | 69.4 ± 19.5 | 66.0 ± 18.8 | 66.4 ± 17.1 | 63.7 ± 20.9 | <0.05 |
| Physical activity (Baecke) | 8.1 ± 3.1 | 8.0 ± 2.9 | 7.8 ± 3.0 | 7.2 ± 3.0 | 0.02 |
| ADL (TMIG–Index) | 11.8 ± 2.1 | 11.7 ± 1.9 | 11.7 ± 2.1 | 11.7 ± 2.1 | 0.93 |
| MMSE | 28.0 ± 2.5 | 28.1 ± 2.4 | 27.7 ± 2.6 | 28.2 ± 2.4 | 0.22 |
| Total energy intake (kcal/day) | 1725 ± 379 | 1732 ± 370 | 1704 ± 330 | 1745 ± 407 | 0.75 |
| Indicated energy amount  (kcal/day) | 1491 ± 193 | 1487 ± 195 | 1487 ± 198 | 1426 ± 187 | <0.01 |
| Protein intake  (g/day/kg BW） | 1.4 ± 0.3 | 1.2 ± 0.2 | 1.1 ± 0.2 | 1.0 ± 0.2 | <0.01 |
| Protein energy ratio (%) | 15.5 ± 2.1 | 15.4 ± 2.1 | 15.6 ± 2.3 | 15.3 ± 2.1 | 0.57 |
| Carbohydrate intake (g/day/kg BW） | 5.1 ± 0.8 | 4.5 ± 0.7 | 4.1 ± 0.6 | 3.7 ± 0.6 | <0.01 |
| Carbohydrate energy ratio (%) | 58.6 ± 5.4 | 59.4 ± 6.4 | 59.3 ± 5.8 | 59.0 ± 5.8 | 0.51 |
| Fat intake (g/day/kg BW） | 1.1 ± 0.2 | 0.9 ± 0.2 | 0.8 ± 0.2 | 0.8 ± 0.2 | <0.01 |
| Fat energy ratio (%) | 25.9 ± 4.4 | 25.2 ± 4.9 | 25.2 ± 4.7 | 25.7 ± 4.6 | 0.30 |

Data are the mean ± SD or n (%)

*P–value for one–way analysis of variance.

BW, body weight; ADL, activity of daily life; MMSE, Mini–Mental State Examination

**Supplementary Figure 1. Survival curve for Cox proportional hazards model**


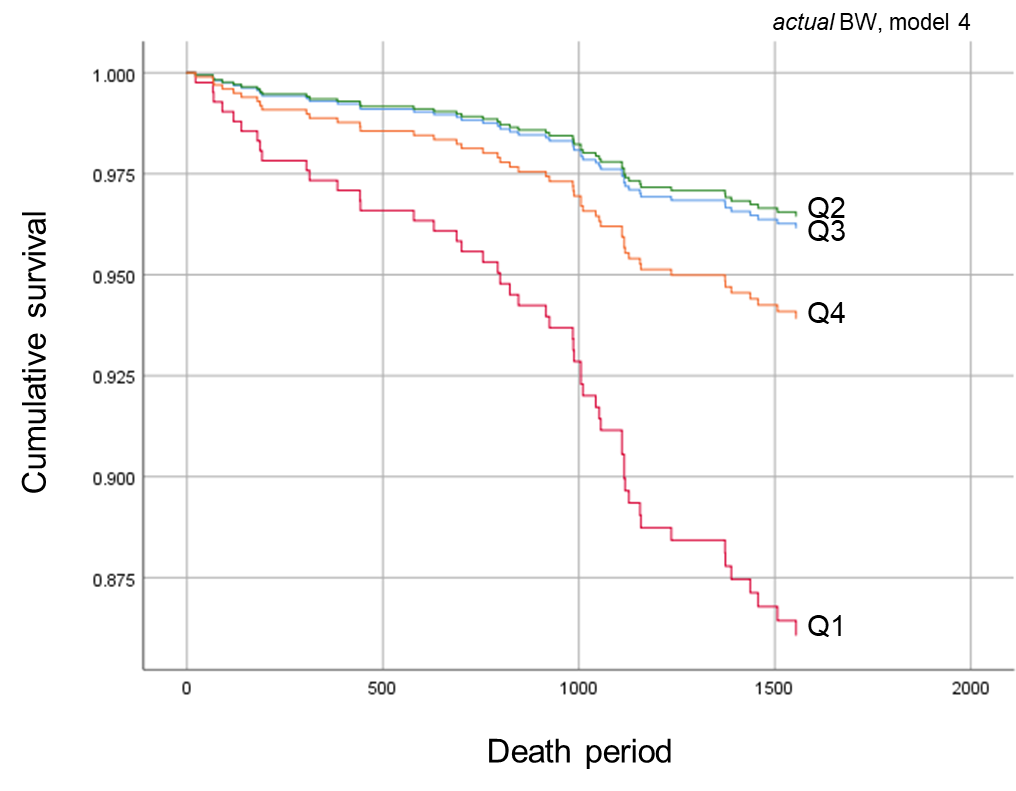

Supplement: Supplementary file 1 — Table S1. Association between dietary content and all‐cause mortality in patients with diabetes mellitus Table S2. Cox regression analysis of quartiles of energy intake per actual bodyweight and all‐cause mortality in model 4, and the model further adjusted for activities of daily living and Mini‐Mental State Examination Table S3.. Baseline clinical characteristics of 756 patients with diabetes mellitus according to quartiles of body mass index Figure S1. Survival curve for Cox proportional hazards model [file GGI-20-59-s001.docx]
